# Development and Pilot Testing of Telesimulation for Pediatric Feeding: A Feasibility Study

**DOI:** 10.1007/s00455-023-10556-3

**Published:** 2023-01-24

**Authors:** Jeanne Marshall, Madeline Raatz, Elizabeth C. Ward, Adriana Penman, Kelly Beak, Madison Moore, Anne E. Hill

**Affiliations:** 1 Speech Pathology Department, Queensland Children’s Hospital, Children’s Health Queensland Hospital and Health Service, Brisbane, Australia; 2 School of Health and Rehabilitation Sciences, The University of Queensland, Brisbane, Australia; 3 Speech Pathology Department, Logan Hospital, Metro South Hospital and Health Service, Brisbane, Australia; 4 Centre for Functioning and Health Research (CFAHR), Metro South Hospital and Health Service, Brisbane, Australia

**Keywords:** Patient simulation, Telepractice, Speech–language pathology, Pediatric feeding disorder, Bottle feeding, Training

## Abstract

Simulation enables learners to practice new skills in a supportive environment. Largely driven by the COVID-19 pandemic, simulation via telepractice, i.e., telesimulation, has emerged. Viable delivery of telesimulation requires consideration of the adaptations needed to conduct simulation via telepractice. The aim of this study was to design and pilot test the feasibility of using telesimulation to provide training in infant feeding management. An iterative process was used across four phases: (1) simulation design, (2) telesimulation adaptations, (3) user testing, feedback, and modifications, and (4) user testing of modified simulation, feedback, and final modifications. During Phases 1 and 2, team members worked together to design and test telepractice adaptations for a simulation experience. During Phases 3 and 4, the telesimulation was pilot tested with a group of speech pathologists, with feedback sought via open-ended survey questions and/or an optional focus group. Manifest content analysis was used to interpret user feedback. In Phase 2, several adaptations were explored to optimize telesimulation delivery and engagement, including Zoom® functions (e.g., ‘spotlighting,’ digital backgrounds) and supplementary video/auditory files. There were 11 participants across Phases 3 and 4. Specific feedback centered around simulation preparation and structure, session practicalities, supports for realism, Zoom® functions, group dynamics, participants’ experiences, and future enhancements. An overall list of recommendations for telesimulation was generated. Telesimulation for feeding management was considered feasible and participant feedback was favorable. Further research is required to investigate if the learner outcomes of telesimulation are comparable to in-person simulation for infant feeding management.

## Introduction

Simulation is widely used in healthcare training and highly regarded for its ability to enable learners to practice new skills in a supportive and safe clinical environment [1–3]. It facilitates active rather than passive participation, by supporting learners to engage in questioning and discussion to supplement their learning [4]. As simulation is traditionally delivered in an in-person modality, distance and travel requirements can impact clinician access to in-person simulation learning opportunities. This was particularly relevant during the recent COVID-19 pandemic when many in-person learning opportunities were suspended.

To help improve access to simulation learning experiences, the concept of telesimulation has recently emerged. Telesimulation is defined as “a process by which telecommunication and simulation resources are utilized to provide education, training, and/or assessment to learners at an off-site location” [5, p133]. Telesimulation as a teaching modality has been explored in medical and nursing education [6–8] and in clinical education settings for allied health students [9, 10]. Results have demonstrated that, in a similar way to in-person simulation [11], speech pathology students accessing telesimulation reported increased confidence and perceived positive impact on clinical skills [9, 12]. Telesimulation was demonstrated to overcome education access barriers imposed due to the pandemic [7, 8] and learners reported high levels of satisfaction and simulation effectiveness [8].

Pediatric feeding disorder (PFD) is estimated to affect one in every 23–37 children under the age of five [13]. The escalating prevalence of PFD [13] has resulted in an increased requirement for clinical skills in this area. Unfortunately, many clinicians report low confidence and readiness to practice [14–16] and expertise is often held at tertiary care centers. Access to training is described as an issue in pediatric feeding care, with time, distance, and travel requirements cited as barriers [16]. A recent Australian survey of training needs found that providing opportunities in an online format was a commonly reported facilitator to increasing access to training, with infant feeding identified as a priority area [16]. In reality, multiple online training opportunities in this clinical area do exist, but most of these are offered using didactic teaching or case-based learning, rather than via an interactive simulated learning experience. Research has demonstrated that content learning is essential as a preliminary step to competence, but that learners require the opportunity to practice and apply their knowledge in a clinical context to consolidate their skills [17, 18]. Simulation offers this opportunity for skills application, critical thinking, and problem-solving [19], and in-person simulation has been demonstrated as a successful facilitator to increase confidence and clinical reasoning skills in pediatric feeding for students [11, 20]. Telesimulation may, therefore, be one solution to previously identified barriers in access to a priority area of training, but this area has not been previously researched in pediatric feeding. The aim of this study, therefore, was to design and test the feasibility of using telesimulation for pediatric feeding training, particularly with regard to an infant feeding case.

## Methods and Results

This study employed an iterative action learning process to develop, trial, and then engage user feedback to evaluate the feasibility of a telesimulation learning experience for pediatric feeding. This process is presented here as four phases: (1) simulation design, (2) telesimulation adaptations, (3) user testing, feedback, and modifications, and (4) user testing of modified simulation, feedback, and final modifications. This study received ethics approval from [name withheld] (HREC/21/QCHQ/80217) and [name withheld] (2021/HE002620).

### Phase 1: Simulation Design

The simulation involved a 3-week-old infant with laryngomalacia and feeding difficulties. Participants worked through information gathering from a nurse, interviewing the parent, completing a feeding assessment and providing recommendations, and providing feedback to the otolaryngologist. The simulation was developed in line with the eleven criteria proposed in the Healthcare Simulation Standards of Best Practice for Simulation Design [21] (Table 1) and in consideration of the barriers and enablers of psychological safety in healthcare simulation [22]. Two of the research team members with expertise in pediatric feeding (Authors 1 and 2) collaborated to design the simulation. Both authors hold a PhD in this topic area, with ten (Author 2) to twenty (Author 1) years of clinical experience. Best practice management for the scenario was derived from literature review regarding infant feeding and laryngomalacia [23–27], as well as the clinical experience of the simulation designers. Consultation was sought with three additional team members with expertise in simulated learning (Authors 3, 4, and 7). These three authors have previously published research in this area, and Authors 4 and 7 hold a PhD in simulated learning. All team members had considerable practical experience with running simulations in an in-person format through multiple years of student and clinician training.

**Table 1:** Alignment of telesimulation components to the Healthcare Simulation Standards of Best Practice™

	Criteria from healthcare simulation standards [21]	Elements of this telesimulation that met best practice standards
1	Simulation-based experiences (SBE) should be designed in consultation with content experts and simulationists knowledgeable in best practices in simulation education, pedagogy, and practice	• Simulation designers had formal and informal training in simulation • Content experts in pediatric feeding had considerable clinical and research experience • Literature review was completed to inform simulation content
2	Perform a needs assessment to provide the foundational evidence of the need for a well-designed simulation-based experience	• A training needs analysis was conducted and identified infant feeding as an area of high need [17]
3	Construct measurable objectives that build upon the learner’s foundational knowledge	• Learning objectives were identified that were specific to the outcomes of the telesimulation (see Table 2)
4	Build the simulation-based experience to align the modality with the objectives	• Telesimulation was designed around key elements (Fig. 1)
5	Design a scenario, case, or activity to provide the context for the simulation-based experience	• Situation and backstory were provided by reviewing a referral and medical notes • A run sheet was developed for consistency and standardization • Progression to the next phase of the simulation was dependent on the learner completing the task (e.g., completing an oral reflex examination)
6	Use various types of fidelity to create the required perception of realism	• A realistic mannequin was used • Costumes and virtual background were changed across different roles • The case provided was developed through literature review and the clinical experience of Author 1 and 2
7	Plan a learner-centered facilitative approach driven by the objectives, learners’ knowledge and level of experience, and the expected outcomes	• Roles were planned during design (one facilitator supported discussions, including debriefing; the other was the simulated nurse/parent/otolaryngologist) • A pause-discuss facilitation approach was planned [23] to support novice-level learners with clinical decision-making in a supported environment • Groups included both novice and experienced participants to support modeling and peer discussion
8	Create a pre-briefing plan that includes preparation materials and briefing to guide participant success in the simulation-based experience	• Pre-briefing session included activities where participants could learn more about each other • Pre-briefing also explored the concept of simulation and session expectations
9	Create a debriefing or feedback session and/or a guided reflection exercise to follow the simulation-based experience	• Planned debriefing was conducted using the method described by Rudolph et al. [24]
10	Develop a plan for evaluation of the learner and of the simulation-based experience	• Feedback regarding learning outcomes and acceptability was sought via survey and focus groups, as part of this research design
11	Pilot test simulation-based experiences before full implementation	• This study is in itself a pilot test

Learning objectives regarding knowledge development, and practical application of assessment, management, and interprofessional communication were developed, targeted at a novice learner with <6-months experience in infant feeding (Table 2). The goals of the simulation were to support learners to develop particular clinical skills (e.g., completing an oral reflex examination) but also to employ critical thinking and problem-solving skills (e.g., making decisions about feeding management), aligning with both behaviorist and constructivist learning theories [19]. Learning opportunities were scaffolded to build on skills and work toward independent practice.

**Table 2:** Learning objectives for the telesimulation

1	Acquire an understanding of feeding and swallowing difficulties in children with laryngomalacia and respiratory distress
2	Conduct a clinical feeding assessment in an infant with laryngomalacia and respiratory distress
3	Evaluate the outcomes of a clinical assessment to identify suitability for oral feeding trials and/or the progression of oral feeding
4	Formulate a management plan using appropriate therapeutic or compensatory strategies
5	Facilitate communication with client/patient, family (caregivers), and other members of the multidisciplinary team.

**Fig. 1:**
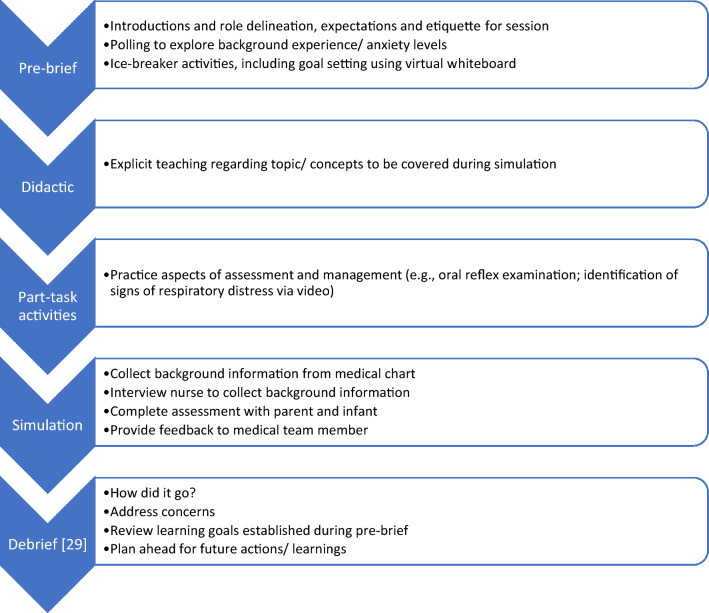
Simulation design

The simulation involved five components as described in Fig. 1: pre-brief, didactic teaching, part-task activities, the simulation itself, and debrief. Pre-learning resources were sent to participants in the form of an online learning module on respiratory distress (Pediatric Feeding Learning Framework, https://ilearn.health.qld.gov.au/d2l/login). This online learning framework was developed as a national resource using a working group of clinicians from across our state and involved literature review, consultation, and external peer review. Other preparatory videos illustrating infant oral reflex assessment, positioning, and feeding equipment were also distributed. The pre-brief session commenced with an ‘ice-breaker,’ which served as an opportunity for participants to establish a psychologically safe learning environment and included personal goal setting for the session. There was time dedicated during the pre-brief to explain the concept of simulation and the expectations for the session, as well as exploring the learning objectives (Table 2). It was made clear during the pre-brief that differences in opinion were welcomed, with the plan that the facilitator would engage in open discussion and present any refuting evidence where necessary. This discussion would be followed up with resources via email after the simulation experience. During the pre-briefing, the roles of the two facilitators were also explained. A brief didactic teaching session was provided to ensure adequate knowledge of laryngomalacia and respiratory distress, prior to commencing the simulation. Part-task activities, where participants practiced specific components of feeding assessment and management, included identifying elements of respiratory distress from a video, reading a vital signs monitor, identifying different equipment for use in the session, and a discussion regarding different management techniques. The simulation itself was designed as a pause-discuss scenario [28], and each participant took a turn at completing a section of the feeding assessment (e.g., nurse interview, parent interview, and infant assessment), with discussion during each pause as to what might happen next and what decisions could be made in assessment and management. Three different ‘scenes’ were presented. This included questioning of the nurse, assessment and management of the infant/mother, and handover to the otolaryngologist. A life-like low-fidelity infant mannequin was used as the ‘patient’, and the ‘mother’ simulated holding and feeding the infant. The session finished with a structured debrief that was facilitator guided [29].

Facilitator 1 (Author 1) acted as the parent/nurse/otolaryngologist, and Facilitator 2 supported discussion and decision-making for the group. Facilitator 1 (Author 1) had considerable clinical experience in pediatric feeding, affording a high degree of content knowledge, which allowed for flexibility in response to participant actions that may have slightly differed from the planned script. As this was planned as a low-fidelity simulation, Facilitator 2 also provided additional auditory cues to support assessment/management during the simulation (e.g., Facilitator 2 would advise participants that the baby was crying, etc.). Before the simulations were run, the facilitators met on three occasions to complete training and troubleshoot any issues (approximately 3 h total).

### Phase 2: Telesimulation Adaptations

Three members of the team (Authors 1, 2, and 3) met on two occasions to troubleshoot translating aspects of the in-person simulation scenario into a virtual medium (via Zoom®). Some guidance was taken from previous literature in medical education [6, 30, 31]. A decision was made to use Zoom® in this study given the accessibility of this platform for most users, enabling easy access and replication of this telesimulation methodology. After exploring the capabilities of Zoom®, various functions were pilot tested with three team members in different rooms at the same physical location to allow for cross-checking.

**Fig. 2:**
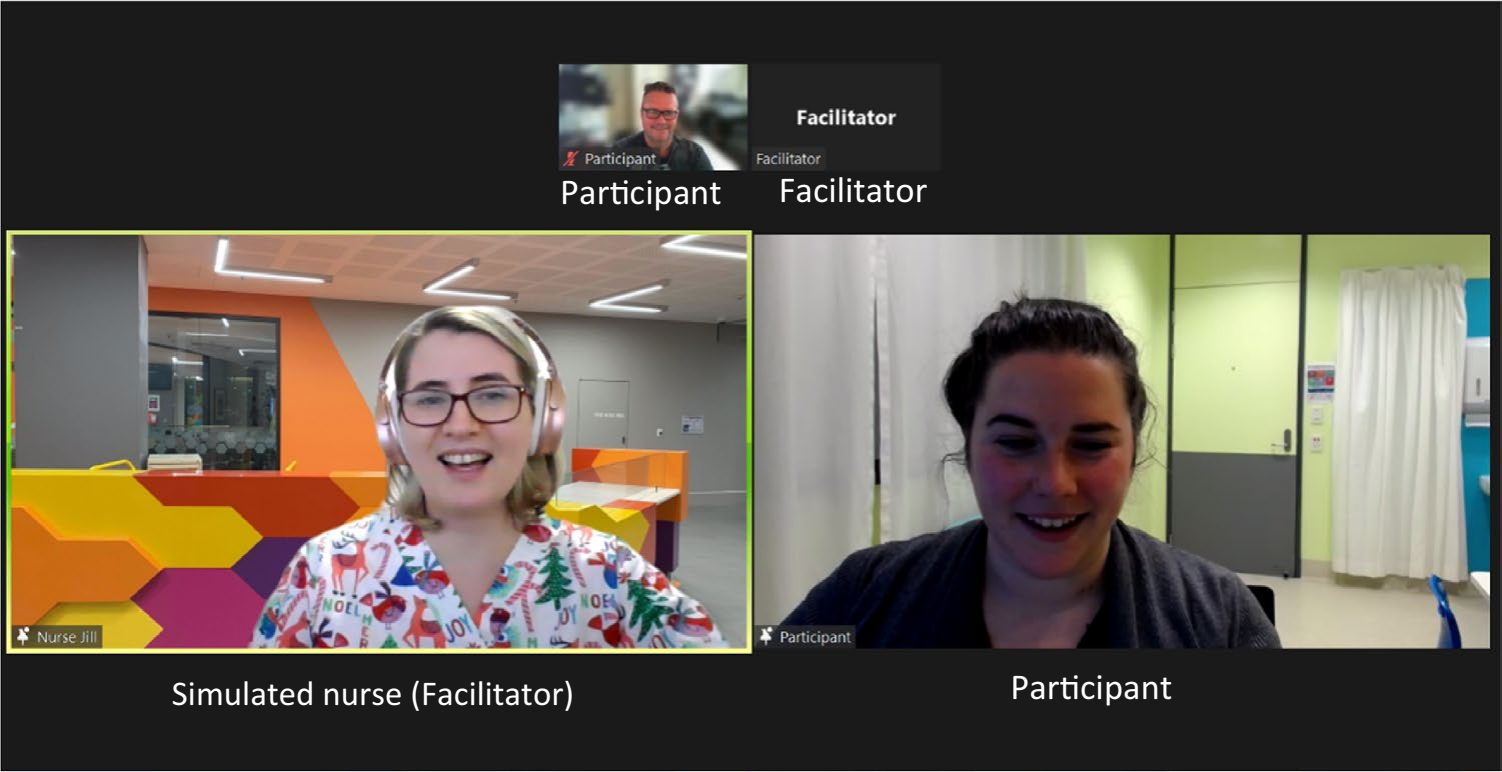
Use of spotlight function to optimize view of simulated nurse (with context-specific background) and participant

**Fig. 3:**
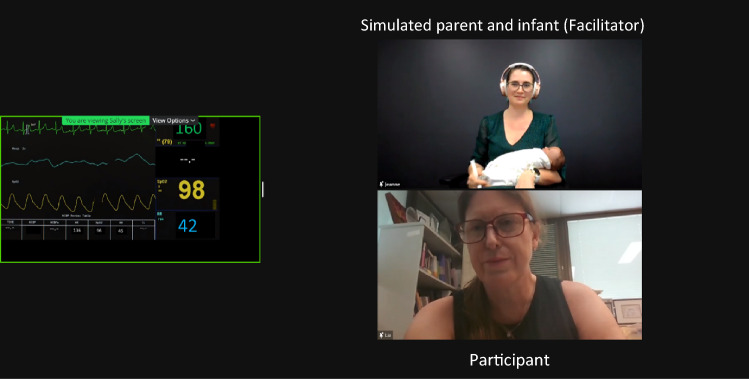
Use of spotlight function enabled displays the simulated parent and infant as well as the “participant” engaging with them and vital signs monitor

It was determined that use of the ‘spotlight’ function to highlight the active participant and the simulated parent/nurse/otolaryngologist would be useful from the participants’ viewpoint (see Fig. 2). The ‘spotlight’ function allows the host to highlight specific Zoom® participants. This makes the spotlighted participants’ tiles larger than those of the other participants, increasing focus on those who are actively engaging during the simulation sequence. The use of different context-specific backgrounds (i.e., clinical ‘scenes’) to support the fidelity of the simulation scenario was also discussed and applied (see Fig. 3). Participants were encouraged to have their view set to ‘speaker view’ during the simulation experience and ‘gallery view’ during the pre-brief and debrief. In speaker view, all learners could be seen in the smaller tiles when only the two participants were spotlighted (Fig. 2), but the other learners could not be seen when the screen was shared with the vital signs monitor (Fig. 3). Zoom® also allowed for manipulation of the size of the Microsoft PowerPoint® screenshared information with the spotlighted participants. Participants were encouraged to reduce the size of the screenshared information and increase the size of the participant tiles by dragging the cursor over the control in the middle of the screen between the two items. It was felt that this manipulation would additionally support ease of viewing and participation (see Fig. 3). The impact of competing noise on the Zoom® platform was also explored, specifically the ability for the simulated nurse/parent/otolaryngologist, participant, and facilitator to all use the microphone simultaneously without impacting on the auditory quality. Team members discussed use of the hand-raise function as a means of coordinating opportunities for participants to contribute.

For the pre-brief section, use of the virtual whiteboard and polling functions within Zoom® was explored in setting up the ice-breaker tasks. The virtual whiteboard was used to brainstorm participant-specific goals for the session, and these were reviewed during debrief at the end of the session. The part-task activities were carefully designed to enable transfer to a virtual medium and included the use of video interpretations, discussion-based tasks, and an equipment sorting activity in breakout rooms with smaller groups. For example, participants watched videos of infants feeding and practiced identifying indicators of respiratory distress with the support of the facilitators. It was determined that it would be valuable for participants to have a doll or plush toy available with them to support skill development in oral reflex examination and for use during the session (i.e., to hold and practice completing an oral reflex exam during the part-task activity).

During the simulation itself, from a physical perspective, the team explored the visibility of the infant/mother and their relative distance from the camera. For the infant/mother portion of the simulation, use of a virtual background impacted on how well the infant mannequin could be seen, so a decision was made to use no virtual background and seat the mother against a dark wall wearing dark clothing. The infant/mother were situated 1.5 m from the camera, on a chair that had a swivel capacity, so that the participants could instruct the mother to move to manipulate their view more easily. Simple costumes (e.g., mother in dressing gown, nurse in surgical scrubs) were used to help support realism for participants (see Figs. 2 and 3). The facilitator’s Zoom® tile label was also changed so to support clarity for participants as to whom they were engaging with.

### Phase 3 and 4: Recruitment for User Testing

All participants for user testing were speech pathologists recruited from local health services (approximately 18-mile radius) using convenience sampling through distribution of an expression of interest. Prior to the session, following the informed consent process, participants were asked to report their experience in infant feeding. For the purposes of this study, participants with <6-months infant feeding experience were considered to be ‘novice’ practitioners, and participants with >6-months experience were considered to be ‘experienced.’ Participants were allocated to either Session 1 or Session 2 based on availability and self-identified experience levels.

### Phase 3: User Testing, Feedback, and Modifications (Session 1)

#### Group Demographics

Pilot testing of the developed simulation program for Session 1 occurred with a cohort of five participants, two of whom were novice and three were experienced (see Table 3). Anonymous polling was used during the ice-breaker section to explore participants’ prior experiences with simulation and feelings of anxiety, with immediate collated feedback provided across the group to support understanding of the perspectives of other participants in the virtual ‘room.’ As can be seen in Table 3, most participants had not participated in a simulated learning experience previously, and the median score (of four participants) for nervousness was 1.5 ranging from 1 (not nervous at all) to 3 (somewhat nervous). Polling data were missing for one participant due to late arrival.

**Table 3:** Demographics of pilot participants

Questions	Responses	Session 1 n (%)	Session 2 n (%)
Clinical experience in infant feeding	< 6-months experience	2 (40%)	3 (50%)
	> 6-months experience	3 (60%)	5 (50%)
Responses to polling questions during ice-breaker section			
Have you ever participated in a simulated learning experience before?	No	3 (75%)	1 (17%)
	Yes, as student	1 (25%)	4 (66%)
	Yes, as a speech pathologist	0 (0%)	1 (17%)
Have you ever participated in a simulated learning experience regarding pediatric feeding before?	No	3 (75%)	3 (50%)
	Yes, as student	1 (25%)	2 (33%)
	Yes, as a speech pathologist	0 (0%)	1 (17%)
On a scale of 1 to 5, how nervous are you feeling about participating in this simulation?	1 = not nervous	2 (50%)	0 (0%)
	2 = a little nervous	0 (0%)	1 (17%)
	3 = somewhat nervous	1 (25%)	3 (50%)
	4 = quite nervous	1 (25%)	1 (17%)
	5 = very nervous	0 (0%)	1 (17%)

#### Group Feedback and Changes

Immediately following the simulation experience, participants were invited to provide feedback via an anonymous survey. Participants were also invited to participate in an optional focus group with a member of the research team not involved in the delivery of the telesimulation, who is an active researcher in the field with a PhD with experience in conducting focus groups and was known to participants (Author 3). Focus groups were conducted virtually via Zoom® and were 20–30 min in duration. As not all participants completed the survey, the same questions were posed during the focus group to allow all participants the opportunity to respond. Additional probes for exploration during the focus group were developed based on responses from the anonymous survey. The survey/focus group guide is provided in Table 4.

**Table 4:** Survey/focus group questions and additional focus group probes

Questions for anonymous survey and focus groups	1. Did this simulation meet your learning needs?
	2. Is there anything in particular you suggest we keep as part of this simulation experience?
	3. Is there anything in particular you suggest we change as part of this simulation experience?
	4. Reflecting specifically on conducting the simulation via telepractice, do you think there was anything that worked particularly well?
	5. Reflecting specifically on conducting the simulation via telepractice, do you think there was anything that did not work well?
	6. Did you experience any technical issues? If yes, please describe them
Additional probes for Focus Group 1	Did you feel you had enough opportunities for discussion during the simulation? What did you think about the size of group involved in the telesimulation?
Additional probes for Focus Group 2	Do you have any comments about the group dynamic and including clinicians with different experience levels in the groups?

Results from the feedback survey and the focus groups were interpreted using manifest content analysis [32] and are reported using guidance from the Consolidated Criteria for Reporting Qualitative Research (COREQ) checklist [33]. Transcriptions were completed by a research assistant and checked by the primary author. Two authors (Authors 1 and 6) reviewed all survey responses and the focus group transcripts and independently developed condensed meaning units and codes. These two authors then met to reach consensus regarding coding and to develop categories from the codes. The full team then met to achieve agreement regarding the findings and resolve any disagreements between Authors 1 and 6. Following this process, a survey was distributed to the participants to determine whether the codes and categories derived were reflective of their experiences and feedback. Five participants responded, with all noting that codes and categories reflected their feedback.

Three participants completed the survey and two participated in the optional focus group (Focus Group 1, identified as Clinicians 1 and 2). As the survey was anonymous, it was not possible to discern whether participants in the focus group also completed the survey. Quotes are presented in the text below with an identifier (e.g., Clinician X). Quotes with no identifier were derived from an anonymous survey response.

Overall, seven categories were agreed upon regarding Session 1 feedback. These included feedback regarding (a) simulation preparation and structure, (b) session practicalities, (c) supports for realism, (d) Zoom® functions, (e) group dynamics, and (f) participants’ experiences of the simulation. Specific coding associated with each of these categories and the learnings applied for Session 2 are presented in Table 5.

**Table 5:** Feedback from participants and changes adopted

Categories	Session 1		Session 2	
	Codes	Learnings for session 2	Codes	Learnings for future simulations
Simulation preparation and structure	• Preparation/pre-learning work important • ‘Ice-breakers helped with comfort/psychological safety • Didactic information before simulation was helpful • Videos were helpful • Part-tasks were helpful before the simulation • Overall flow of simulation good		• Preparation/ pre-learning work before simulation important • ‘Ice-breakers helped with comfort/psychological safety • Overall flow of simulation good • Reviewing goals at the end helpful • Pre-brief important	
Session practicalities	• Not enough time for overall simulation • More breaks needed • More time needed for discussion regarding patient management • Not enough time for debrief • Facilitator roles were explained and clear • Pause-discuss format worked well • Participants went to breakout room before hearing instructions • Internet outage resulted in difficulty with access to Zoom®	• Allocate more time for the simulation, allowing for breaks • Spend more time discussing different management options during case • Explain activity before commencing breakout room • Ensure contact details are provided for participants experiencing any technical issues	• Not enough time for debrief • Pause-discuss format worked well • Needed more context for case	• Allocate more time to debrief • Continue use of pause-discuss format • Provide more context for case
Supports for realism	• Acting was good • Backgrounds helpful • Costumes helpful • Mannequin realistic • Vital signs monitor helpful • Not enough information from facilitator/audio to fully participate in assessment task	• Provide recording of stridor in infant during simulation • Facilitator to provide more auditory assessment points (e.g., “*the infant is having an apnea now”)*	• Acting was good • Costumes helpful • Too many things happening at once • Poor audio quality for stridor • Need to balance facilitator commentary with realism	• Careful scripting/rehearsal of facilitator cueing • Limit auditory information to facilitator cues only
Zoom® functions	• Spotlight function works well • Hand-raise function not used enough	• Explain and reiterate use of hand-raise function	• Hand-raise function used well • Virtual whiteboard worked well • Variety of participation options (e.g., chat, whiteboard) helpful	
Group dynamics	• Small group size was good • Modeling from others was helpful • Skill mix good for learning • No immediate supervisor in group promoted psychological safety	• Consider relationships in group for psychological safety	• Small group size was good • Modeling from others was helpful • Skill mix good for learning • Novices and experienced clinicians should be split	• Keep groups to approximately 6 participants • Provide further preparation for novice participants regarding expectations for simulation
Participants’ experiences of the simulation	• Case easy for experienced clinician but challenging for novice clinician • Participant stressed during simulation • Good headspace during simulation • Comfortable to participate • Zoom® was convenient and easy to access	• Ensure there are adequate supports in place for participants to pause/take a break	• Comfortable to participate • Inclusive learning experience • Expectations were met • Lack of confidence to volunteer • Uncomfortable to participate Simulation increased confidence • Simulation consolidated knowledge • Self-consciousness with camera • Telehealth reduces barriers to learning	• Provide further preparation for novice participants regarding expectations for simulation • Make participants aware that they will be able to see themselves
Future enhancements			• For future simulations could add increased social complexity to cases • For future simulations could extend the case over multiple sessions • Split the group into separate breakout rooms for activities dependent on skill mix • Include more peer discussion regarding problem-solving	

In *simulation preparation and structure*, participants reported that the pre-learning activities “set up the course well” and one participant felt that “…if you hadn’t had done that first or even had that theoretical refresher with [Facilitator 1] before getting into the SIM [telesimulation], it would have been really challenging…” (Clinician 1). Participants also appreciated the ‘ice-breakers’ at the beginning of the session and commented that they helped them feel that “others were in the same boat” and that “they’re safe people” (Clinician 2). Participants enjoyed that the activities were layered to build up to simulation participation, i.e., “you sort of built up…you had these little practical tasks along the way prior to getting into the full-blown sim…” (Clinician 1). The overall flow of the simulation was considered to be appropriate by multiple participants.

Regarding *session practicalities*, multiple participants reported that time was an issue during the session. Specific areas for improvement included allowing adequate breaks, increased time for discussion of management options during the simulation, and increased time for the debrief. One participant suggested “it would also be helpful just to have more time to sit to pause and discuss what is the rationale for [that]?” (Clinician 2). This feedback resulted in the extension of Session 2 by one hour. There was also an issue with breakout room initiation, where participants left to join a breakout room prior to having the activity explained to them. This was remedied for Session 2 by not initiating breakout rooms until after the activity description had been completed. Finally, on the day of Session 1, there was a state-wide internet outage, which impacted participants’ ability to join into the Zoom® session at the scheduled start time. This resulted in some participants being late to join in, there was a malfunction of the breakout room function, and some lag with Microsoft PowerPoint® sharing, which caused the session to run slightly late. Participants recognized these issues noting that the “only tech issues were from the…internet.” The team discussed ensuring that contact details are provided to support troubleshooting in the event that participants may be experiencing any technical issues.

Participants described that the backgrounds, costumes, vital signs monitors, and the “real-looking doll” were all helpful *supports for realism* during the simulation. One participant reported “[Facilitator 1]’s acting was really good and it actually felt very realistic. It made me feel like I was in that clinical situation with how stressed she was…” (Clinician 1). Several participants described that there was not enough auditory information (i.e., infant respiratory sounds) to participate in the initial assessment with the mother/infant. One participant described their experience:

I was sort of holding back and waiting for that [auditory information]. But in waiting for that I was seeing the monitor go up and then getting quite anxious that I wasn’t doing anything. Um, so that was just something that I had to overcome, be like, okay, well, we need to stop, obviously. (Clinician 2)

Subsequently, a decision was made to add recordings of infant stridor sounds and more auditory assessment points from Facilitator 2 for Session 2.

Regarding the *Zoom® functions*, participants reported that use of the ‘spotlight’ function in Zoom® meant that “only the clinician role-playing and the presenter were viewed—this really helped to ‘get in to character’ and act like we usually would in a work scenario.” Although the facilitators had intended to use the ‘hand-raise’ function during the session, this was not used consistently, and several participants reiterated statements regarding preferring to have used the hand-raise function, for example, “to make sure that I’m giving other people opportunity… I would have appreciated maybe more consistent use of the hands up…” (Clinician 1). Subsequently, the facilitators planned to more clearly explain and reiterate use of the hand-raise function for Session 2.

*Group dynamics* were an important consideration in setting up the simulation experience to support psychological safety. Participants fed back that the group size of six participants was good, allowing participants to “share ideas/opinions with comfort,” get a “decent go at doing the practical tasks” (Clinician 1), and to “learn and to grow and also make mistakes” (Clinician 2). Having a mixture of experienced clinicians and novice clinicians was viewed as valuable, but it was important to identify different levels of experience early in the session so that the participants are “all on a level playing field” (Clinician 2).

Yeah, I was, I was glad that there were some other people that were fairly new to, like Ped [pediatric] feeding clinical work as well. So I think it’s important to have not just one person who’s the newie (Clinician 2).

One participant identified that not having any direct supervisors in the group was helpful to her “comfort” and that “if one of my supervisors was there as well, it might then create a bit more anxiety if I were to make a mistake while learning in that space” (Clinician 2).

In general, *participants’ experience* of the simulation put them in a “good headspace.” The case was considered to be “bread and butter” for an experienced clinician but challenging for a novice. One participant reported experiencing some stress during the scenario, although this was more to do with the authenticity of the set up than the simulation experience itself. Participants reported the simulation was “convenient and easy to access” via Zoom®.

### Phase 4: User Testing of Modified Simulation, Feedback, and Final Modifications (Session 2)

#### Group Demographics

Following user feedback, modifications were made to the program and a second set of clinicians completed the revised simulation program. This group included six participants, three of whom described themselves as novice practitioners and three of whom were experienced. More participants from Session 2 had participated in simulation experiences previously, although this was predominantly during undergraduate study. The median score for nervousness was 3, ranging from 2 (a little nervous) to 5 (very nervous) (Table 3).

#### Group Feedback and Changes

Four participants completed the anonymous survey following the telesimulation and four (identified as Clinicians 3–6) participated in the optional focus group (Focus Group 2). Data from both the survey and the focus groups were interpreted using manifest content analysis. Again, six categories emerged, including (a) simulation preparation and structure, (b) session practicalities, (c) supports for realism, (d) Zoom® functions, (e) group dynamics, and (f) participants’ experiences of the simulation with the addition of a new category, “future enhancements” (See Table 5).

Regarding *simulation preparation and structure*, this second set of participants provided similar positive feedback to the clinicians who took part in the first telesimulation session, with several participants emphasizing the importance of the pre-brief in making participants “feel comfortable” (Clinician 5) and setting the expectation that “this was a learning opportunity and not an assessment.” When considering the debrief, participants reported that having the opportunity to “reflect at the end on what I learnt/gained from the experience” was helpful. With regards to *session practicalities*, participants reported again that they benefited from the pause-discuss format of the simulation, but that there was not enough time for an adequate debrief at the end of the session.

In response to feedback from Session 1 and to facilitate *supports for realism*, audio clips depicting stridor in the infant were played and increased facilitator cueing was added into the second presentation of the telesimulation learning experience. This, however, resulted in excessive audio-visual activity, which was reported as “distracting” by the participants. The audio quality of the stridor sounds was also described as poor, with one participant commenting, “The sound to me kind of sounded like a cough, but it was actually stridor.” (Clinician 4). As such, the team made the decision to limit auditory information to facilitator cues only for the future and to complete careful scripting and rehearsal of this cueing. Participants again described the overall experience as realistic, commenting particularly on the acting and costumes of Facilitator 1.

In *Zoom® functions*, participants fed back that the “hand-raising function worked well to ensure no one spoke over the top of each other” in this session. Additionally, participants enjoyed use of the virtual whiteboard function in Zoom® and felt that the “multiple methods of communication (writing in chat, speaking on camera, breakout groups) gave different participants a chance to contribute in a way they preferred,” which was identified as an additional benefit of the telesimulation modality. One participant described feeling that the telesimulation modality allowed them to participate more equally than if they were in-person:

Yeah so, one thing that I thought was a great advantage of the telehealth setup was that we were all very much even, it was a very equal experience. Because typically, you know, you walk into a room where potentially a group are quite familiar with each other and they might sit next to, you know, one another, if they’re already well known. And equally, there’s a bit of an ordering in terms of your approximation to the person presenting, etc. And so the fact that we can all visibly see each other, very equally, I thought was a real advantage. (Clinician 3).

This same participant commented on the disconcerting nature of the camera, describing that “when you can actually see yourself the whole time, I think it does add an extra level of intensity and an added level of self-consciousness perhaps” (Clinician 3).

The *group dynamics* for Session 2 were different to Session 1. Despite there being more novice participants in Session 2, two novice participants felt uncomfortable to participate, reporting that they “…would have felt more comfortable in a group with other more novice speechies [speech pathologists] in this area” (Clinician 6) and suggesting that novice and experienced clinicians should be split. These clinicians described “feeling inferior to the other clinicians in the group who work with feeding on a daily basis.” Conversely, other participants in the group “loved having a mix, because it created a bit of a safe space” (Clinician 3). These participants described the value of having different perspectives in the group, stating things like “…actually if there are people going back to the basics, asking pretty simple questions, it makes it safer to then be a bit more vulnerable in the learning process, I think” (Clinician 3). Multiple participants also valued the modeling of clinical skills that more experienced clinicians could provide. The research team discussed this issue at length and decided that the benefit of incorporating a group with varied skill mix outweighed the risks in this scenario, but that further preparation opportunities should be provided to novice participants so that they know what to expect during the simulation experience.

Skill mix issues did influence the report of *participant experiences*, where some participants reported feeling uncomfortable and a lack of confidence to volunteer, describing that “they were happy to just sit and observe and learn” (Clinician 5). Other participants in the group, however, reported positive experiences, including that the simulation increased their confidence and consolidated their knowledge, describing it as a “rich learning experience” (Clinician 3) that “met my expectations.” One participant reported that the format of learning for them was more accessible:

“I accessed this learning experience without having to leave my worksite and therefore feel that the telehealth format, for me, reduces some barriers to learning (requesting leave, increased travel time to and from learning facility).”

Finally, this group raised several suggestions for *future enhancements* for the telesimulation experience, including that future cases could include increased social complexity or be conducted over multiple sessions (i.e., the simulation might involve an initial assessment and multiple reviews for the same patient). Further opportunities for peer discussion regarding problem-solving and case management, particularly about “what you wouldn’t do or when things don’t go to plan” (Clinician 3) were reported as suggested future inclusions. Finally, split breakout rooms between novice and experienced clinicians for part-task sessions were suggested to support more targeted discussions at similar skill-mix levels.

A final list of general recommendations for telesimulation based on these findings is presented in Table 6.

**Table 6:** Final list of general recommendations for telesimulation based on findings

Zoom® platform	• Use Zoom® functions including spotlight, virtual whiteboard, and background changes • Use hand-raise function for participant discussion • Ensure activities for breakout rooms are explained prior to initiating breakout room sequence • Ensure contact details are provided for participants experiencing any technical issues
Group dynamic	• Keep group sizes to 5–6 participants • Carefully consider group dynamic with regards to impact on psychological safety • Include all levels of experience, but suggest extra pre-learning for novice participants • Ensure all participants are aware of what the simulation will involve before they log in
Facilitators	• Maintain separate roles for facilitator vs. simulated patient/nurse/parent • Consider using subject matter expert as simulated patient/parent
Pre-learning	• Include relevant pre-learning activities for all participants to prepare them for the content in the simulation • Include extra pre-learning for novice participants
Ice-break activities	• Include polling to support anonymous declaration of experience/confidence/anxiety to allow the group to feel safe with each other from the outset • Work with the group at the beginning to set personal learning objectives for the session
Pre-brief	• Ensure participants understand this is not an assessment, and they are not forced to participate
Simulation	• Avoid too many competing sounds during active simulation • Use facilitator to introduce concepts verbally that may not be obvious visually
Debrief	• Allow at least 20 min for debrief • Suggest reviewing personal learning objectives set at the beginning of the session
Time management	• Allow at least 4 h per simulation to provide adequate time for pre-briefing, content, and debriefing, as well as adequate time for discussion/clinical problem-solving

## Discussion

This research used an iterative approach across four phases to design, pilot test, and refine an infant feeding management telesimulation. Previous research in telesimulation has predominantly explored training and development for medical and nursing staff [6–8, 30, 31]. Although many learnings can be taken from this previous work, to our knowledge, this is the first study to explore the use of telesimulation in pediatric feeding management in speech pathology, apart from in a clinical education setting with university students [9, 10]. Overall, it was concluded that telesimulation was a feasible teaching modality for infant feeding management, and participant feedback was generally positive.

The outcomes of this research suggest that telesimulation may be a solution to providing practical training opportunities for clinicians that may not otherwise be able to access these due to geographical separation. Other studies in this area have also supported the feasibility of telesimulation for remote participants [34, 35]. It is argued, however, that the convenience of telesimulation may also improve access to professional development for all clinicians, regardless of physical location. As described by one participant in the current study who was working in a metropolitan setting close to where the facilitators were located, telesimulation allowed them the chance to attend the professional development event without leaving their workplace, having to account for travel time, or organize leave. Telesimulation may have further applications in pediatric feeding that should also be investigated, including increased opportunities for skills maintenance, complex case management, and best practice alignment for experienced clinicians and peer discussion, permitting networking and collaboration across different facilities. Preliminary data also suggest that the costs associated with this education modality are lower than those incurred for in-person training [5, 35]. The accessibility and potentially lower cost of telesimulation may facilitate the provision of more professional development opportunities to clinicians, which may have a direct impact on service provision and care outcomes for children with PFD, regardless of clinician location.

There were several technical features adopted during the adaptation process that were considered to be critical to the success of transitioning simulation to the telepractice modality, including changing backgrounds and tile labels, screen sharing, use of the spotlight function, polling, the virtual whiteboard, and hand-raise function. The preparation sessions with the facilitators during telesimulation design were essential in developing familiarity with the different Zoom® functions. Use of the ice-breaker sessions during the first part of the simulation also allowed the participants to familiarize themselves with the view, mute, and hand-raise buttons. This allowed the facilitators and the participants to focus on the activity instead of the Zoom® logistics during the simulation itself. Ensuring participants had a doll or teddy-bear available locally also supported practical activities, such as oral reflex examination and positioning. Future adaptations could include sending preparatory materials for participants regarding the Zoom® platform itself [31].

Multiple participants commented on the authenticity of the simulation, which was contributed to by the use of virtual backgrounds and costumes, the screen sharing of the vital signs monitor, and the “quality of the acting.” Visual supports including the backgrounds, costumes, and monitor were specifically designed to replicate those that might be observed in a clinical environment. Use of a simulation-trained clinician and content expert to act as the parent allowed for spontaneity and flexibility in response to the simulation direction (i.e., the content expert can be responsive to subtle directions the simulation takes, rather than relying on a purely scripted response). This increases the realism and authenticity of the experience for participants and is suggested as the reason for perceived high-quality acting. It is known that clinical accuracy of the simulated patient contributes to the fidelity of the simulation and is important for learner outcomes [36]. This provides further evidence for the importance of using correctly trained simulated patients to ensure authenticity during simulation experiences.

There were differences in group dynamics between the two sessions. In Session 1, participants indicated that they enjoyed the variation of clinician experience within the group, as it allowed for a feeling of safety for novice participants (i.e., “I’m not alone”), and experienced participants were able to act as models for learning and consolidate their knowledge, creating a peer learning opportunity. Certainly, this has been the experience of the research team in previous in-person simulations. In Session 2, however, two participants did not feel comfortable to engage, as they felt they did not have enough baseline knowledge/experience to contribute. It is unclear why these novice participants were different to those in Session 1 in terms of comfort to participate, but it was clear that they did not feel psychologically safe enough to engage. Given simulation is new to many speech pathologists, it was felt that further information regarding structure and content prior to the simulation for these participants may have been helpful to prepare them with regards to expectations and readiness to engage. Distributing a telesimulation agreement prior to the session with details regarding confidentiality and learning expectations is in line with recent telesimulation literature [31]. Finally, using the pre-brief to ensure the group are familiar with the experience levels of different group members and the expectations for these different experience levels during the simulation is essential to ensuring adequate psychological safety to participate.

### Limitations

It is acknowledged that this simulation only catered for speech pathologists and was targeted at a specialized area of speech pathology care. Additionally, this pilot work only included a small sample size. Future studies could explore application of telesimulation across multidisciplinary teams and/or across different areas of speech pathology practice.

## Conclusion

Telesimulation for infant feeding was considered to be feasible, and participants reported generally positive experiences. Adequate preparation, familiarity with the various technological enhancements, small group sizes, and thorough pre-briefing were important success factors for telesimulation. Further exploration as to whether learners can achieve the same outcomes in telesimulation as compared to in-person simulation is now required.
